# MiR-126 in intestinal-type sinonasal adenocarcinomas: exosomal transfer of MiR-126 promotes anti-tumour responses

**DOI:** 10.1186/s12885-018-4801-z

**Published:** 2018-09-17

**Authors:** Marco Tomasetti, Massimo Re, Federica Monaco, Simona Gaetani, Corrado Rubini, Andrea Bertini, Ernesto Pasquini, Cristiana Bersaglieri, Massimo Bracci, Sara Staffolani, Mariastella Colomba, Armando Gregorini, Matteo Valentino, Adriano Tagliabracci, Massimo Bovenzi, Jiri Neuzil, Monica Amati, Lory Santarelli

**Affiliations:** 1 Department of Clinical and Molecular Sciences, Section of Occupational Medicine, Polytechnic University of Marche, Via Tronto 10/a, 60020 Ancona, Italy; 2 International Society of Doctors for the Environment (ISDE), Arezzo, Italy; 3 Department of Clinical and Molecular Sciences, Section of Otorhinolaryngology, Polytechnic University of Marche, Ancona, Italy; 4 Department of Biomedical Sciences and Public Health, Section of Anatomical Pathology, Polytechnic University of Marche, Ancona, Italy; 5 Surgical Department, ENT Metropolitan Unit, Bellaria & Budrio Hospital, Bologna, Italy; 6 Department of Biomolecular Sciences, University of Urbino “Carlo Bo”, Urbino, PU Italy; 7 Department of Biomedical Sciences and Public Health, Section of Legal Medicine, Polytechnic University of Marche, Ancona, Italy; 8 Department of Medical Sciences, Clinical Unit of Occupational Medicine, School of Medicine, University of Trieste, Trieste, Italy; 9 School of Medical Science, Griffith University, Southport, Australia; 10 Institute of Biotechnology, Czech Academy of Sciences, Prague-West, Czech Republic

**Keywords:** Intestinal-type sinonasal adenocarcinomas, Sinonasal-inverted papillomas, Inflammatory polyps, Biomarker, Exosomes, MiR-126, MiR-based therapy

## Abstract

**Background:**

Intestinal-type sinonasal adenocarcinomas (ITACs) are aggressive malignancies related to wood dust and leather exposure. ITACs are generally associated with advanced stage at presentation due to the insidious growth pattern and non-specific symptoms. Therefore, biomarkers that can detect the switch from the benign disease to malignancy are needed. Essential for tumour growth, angiogenesis is an important step in tumour development and progression. This process is strictly regulated, and MiR-126 considered its master modulator.

**Methods:**

We have investigated MiR-126 levels in ITACs and compared them to benign sinonasal lesions, such as sinonasal-inverted papillomas (SIPs) and inflammatory polyps (NIPs). The tumour-suppressive functions of MiR-126 were also evaluated.

**Results:**

We found that MiR-126 can significantly distinguish malignancy from benign nasal forms. The low levels of MiR-126 in ITACs point to its role in tumour progression. In this context, restoration of MiR-126 induced metabolic changes, and inhibited cell growth and the tumorigenic potential of MNSC cells.

**Conclusions:**

We report that MiR-126 delivered via exosomes from endothelial cells promotes anti-tumour responses. This paracrine transfer of MiRs may represent a new approach towards MiR-based therapy.

**Electronic supplementary material:**

The online version of this article (10.1186/s12885-018-4801-z) contains supplementary material, which is available to authorized users.

## Background

Cancers of the nasal cavity and the paranasal sinuses, referred to as sinonasal cancers (SNCs), are associated with occupational exposure to wood, leather and nickel particles that are reported as risk factors for the development of these tumours [1]. SNCs account for 0.2% of all primary malignant neoplasms, with an incidence of 0.1–1.4 new cases/year/100.000 subjects. Around 8–25% of all SNCs are intestinal-type adenocarcinomas (ITACs), which are malignant epithelial tumours related to wood dust exposure. This type of cancer is characterized by frequent local recurrences, low incidence of distal metastases, and overall mortality of approximately 53%. The clinical stage and histology of the tumour have a considerable impact on survival. Surgery with or without radiotherapy represents the preferred treatment of these tumours. In spite of aggressive surgery and high-dose radiation therapy, the low rate of long-term survival of patients with SNC is disappointing [2]. Certain benign forms of the sinonasal pathology, such as sinonasal-inverted papillomas (SIPs), can progress to sinonasal squamous cell carcinoma (SNSCC), and the incidence of the malignant switch in individual series of SIPs was reported as 5–10% [3, 4].

The molecular mechanism involved in the pathogenesis of ITACs is largely unknown [5, 6]. Chronic inflammatory states and angiogenesis have been linked to the development of malignancy [7]. Angiogenesis is an important process in tumour progression, the vascular endothelial growth factor (VEGF) being one of the key drivers of both physiological and pathological blood vessel formation. VEGF, as well as angiomotin, a protein that regulates tubule formation, are significantly increased in SIP, which was suggested to contribute to its progression by promoting angiogenesis [8, 9]. Angiogenesis has been also related to the pathogenesis and prognosis of chronic rhinosinusitis with nasal polyposis [10]. Nasal inflammatory polyps (NIPs) include mucosal epithelial hyperplasia, infiltration of inflammatory cells, neo-vascularization, and edema [11].

Both angiogenesis and inflammation are processes regulated by microRNAs (MiRs), such as MiR-126, which is encoded by intron 7 of the epidermal growth factor-like protein 7 (*EGFL7*) gene [12]. MiR-126 is mainly expressed by endothelial cells (ECs), and plays a critical role in several human cancers [13]. It has been shown that MiR-126 inhibits tumour growth by prevention of cancer cell proliferation, migration, and invasion, but also by down-regulation of central signal molecules of cancer cell survival [14–16]. To evaluate the role of MiR-126 in the malignancy of SNCs, expression and distribution of MiR-126 were investigated in malignant ITACs and compared to sinonasal benign neoplasms, such as SIPs and NIPs. The tumour suppression function of MiR-126 was also evaluated, supporting its role as a potential novel cancer suppressor.

## Methods

### Specimens

Fresh and formalin-fixed paraffin-embedded (FFPE) tumour tissues and matched adjacent normal epithelium were obtained from 58 surgically treated patients; 23 of them were diagnosed with ITACs, 15 with SIPs, and 20 had documented NIPs. All samples were collected from the archive of the Pathological Anatomy Unit of the Hospital University of Ancona, and from the Otorhinolaryngology Department of Budrio-Metropolitan Hospital Bologna, Italy. The clinical data included information on gender, age, histology, staging according to the TNM classification (UICC 2009), therapeutic protocol and the follow-up conditions.

Whole blood was collected from ITAC patients (*n* = 13) at the time of clinical examination and serum was prepared. The control group consisted of 19 healthy subjects recruited at the clinical of Occupational Medicine, Polytechnic University of Marche, Ancona, Italy. None of them had ever been occupationally exposed to wood and leather dust, as documented by their occupational histories.

### RNA, circulating MiR-126 isolation and quantitative RT-PCR

Total RNA from cultured cells and tissue samples (10–100 μg) was obtained using the RNeasy Mini Kit (Qiagen) and the RecoverAll total nucleic acid isolation kit (Ambion), respectively, according to the manufacturer’s instructions. MiR-126, IRS1, VEGF-A, SOX-2 and EGFL7 first-strand cDNA was synthesized using the High-Capacity cDNA Reverse Transcription Kit (Applied Biosystems). Quantitative RT-PCR (qPCR) was performed using the TaqMan Gene Expression Master Mix (Applied Biosystems) with U6 and GAPDH as the housekeeping gene for the MiR and the genes, respectively. qPCR assays were performed using the Mastercycler EP Realplex instruments (Eppendorf) and results expressed as relative level (2^-ΔCT^), and fold changes (2^-ΔΔCT^). For circulating MiR-126 detection, RNA was isolated from 250 μl of diluted serum (1:10), or from the exosomal fraction (20 μg protein) and MiR-126 was detected as previously described [17, 18].

### Bisulphite modification and genomic sequencing

The methylation status of CpG dinucleotides close to the *EGFL7* T2 promoter was analysed. The bisulphite sequencing assay was performed using 1 mg of bisulphite-treated genomic DNA from malignant tissue and adjacent non-cancerous tissue of seven patients with SNC. Bisulphite conversion was performed using the EZ DNA Methylation™ Kit (Zyno Research, Euroclone) according to the manufacturer’s instructions. Fragments of interest were amplified using the following specific primer pairs designed with the Primer3 software, i.e. forward: 5′-TGA TTT AGT GAT TTC GGT GAG G-3′; reverse, 5′-AAC CCT TTA CTA ACT TTC AAA CCC-3′. PCR products were gel-purified by means of the Wizard SV gel and PCR Clean-up kit (Promega) or the FastGene Gel/PCR Extraction Kit (Nippon Genetics), and sequenced using the Reverse primer (5′-AAC CCT TTA CTA ACT TTC AAA CCC-3′) to analyse the DNA methylation status. Sequencing of purified PCR products was carried out using automated DNA sequencers at Eurofins MWG Operon (Germany). All sequences were visualized with BioEdit Sequence Alignment Editor 7 [19] and aligned with the ClustalW option included in this software.

### Cell culture

Malignant nasal squamous cell carcinoma from the pleural effusion (MNSC, RPMI 2650) and fibroblasts (IMR-90) were obtained from the ATCC and grown in the RPMI-1640 and DMEM medium, respectively, with 10% FBS, 1% penicillin and 10% streptomycin (Life Technologies). Human umbilical vein endothelial cells (HUVECs) obtained from GIBCO (Life Technologies) were grown in Medium 200 with the large vessel endothelial supplement (LVES, Life Technologies). Cells were maintained at 37 °C and 5% CO_2_, and cultured for not more than six passages within 1 month after resuscitation and periodically checked for the absence of mycoplasma contamination using the PCR Mycoplasma Test. Cell authentication was performed using the PowerPlex Fusion 6C System (Promega, Fitchburg, WI).

### Exosome isolation and exosome uptake

Exosomes were isolated from HUVECs cultured in exosome-depleted serum-containing medium as previously described [18]. For exosome uptake assay, isolated exosomes were stained with PKH-67 (20 μM, 4 min; Sigma), a probe used to label lipids on membrane surface of exosomes. After washing in PBS, PKH-26-labelled exosomes were placed in conditioned medium of MNSC cells, and uptake was periodically analysed by flow cytometry (FACS Calibur, BD). Alternatively, uptake was assessed after 4 h incubation by fluorescent microscopy (Axiocam MRc5 Zeiss).

### Ectopic MiR-126 expression

MNSC cells (2 × 10^4^ per well in a 24 well plate) were stably transfected with the pCMV-MiR plasmid carrying the MiR-126 sequence 5’-UCG UAC CGU GAG UAA UAA UGC G-3′ (OriGene) using the TransIT-LT1 reagent (Mirus). Selection of transfected cells was performed using G418 (0.6 mg/ml; Sigma) added to the cell culture medium after transfection. G418-resistant clones were analysed for MiR-126 expression and maintained in the RPMI media with 0.6 mg/ml G418. Alternatively, cells were transiently transfected with the MiR-126 mimetic (MISSION® microRNA Mimic; Sigma) using High Perfect Transfection reagent (Qiagen). MiR-126 function was blocked with the anti-sense oligonucleotide 5’-GCA UUA UUA CUC ACG GUA CGA-3′ (IDT); scrambled sequences were used as a control.

### Acid vesicles and red oil O staining of lipids

Acid vesicles (AVs) in MNSC cells and their MiR-126-transfected counterparts were evaluated by acridine orange (AO) staining. Cells were seeded on coverslips in a 6-well plate, allowed to attach overnight, incubated with 5 mg/ml AO for 30 min at 37 °C. For detection of lipid droplets (LDs), cells grown on coverslips were fixed in 70% (*v*/v) cold ethanol and stained with the Oil Red O solution in 60% (v/v) isopropanol. Cells were analysed using fluorescent and optical microscopy, respectively (Zeiss, Axiocam MRC5; magnification 40× or 60×).

### Migration and cell proliferation assay

Migration assays were performed after seeding cells in the 12-well plate allowed to reach confluence and treated with mitomycin-C (5 μg/ml, 3 h; Sigma) to block the cell cycle. Cells were wounded by scratching with sterile pipette tip and supplemented with exosomes isolated from HUVECs (20 μg/ml). Migration was quantified as wound-healing percentage of the cells determined by the ratio of the ‘wound width’ at 72 h time and the wound width at 0 h. The MTT assay (Sigma) was performed and Ki67-positive cells were estimated as a measure of cell proliferation. MNSC cells and the MiR-126 plasmid- or mimetic-transfected cells (3 × 10^4^ cells/well in 96-well-plate) were incubated at different time points and cell viability evaluated. Alternatively, cell viability was analysed in MNSC cells exposed to exosomes isolated from HUVECs. Ten microliters of MTT (5 mg/ml) was added to each well and after 3 h incubation, the crystals prodused were dissolved in isopropanol and absorbance read at 550 nm in an ELISA plate reader (Sunrise, Tecan). The results were expressed as relative change with respect to the controls set as 100%. Proliferation was assessed in permeabilized cells using the anti-Ki67 IgG and fluorescence microscopy (Zeiss; Axiocam MRc5, magnification 60×). The proliferation index was expressed as a percentage of Ki67-positive cells.

### Glucose uptake and glucose, ATP and lactate detection

MNSC cells were seeded in 96-well black-bottom plates (3 × 10^4^ cells per well) in low glucose (1 g/l) DMEM at 5% CO_2_ and 37 °C. After overnight incubation, the cells were treated with 2-nitrobenzodeoxyglucose (2-NBDG, 50 μM) for 30 min. The level of fluorescence intensity was evaluated at 550/590 nm using a fluorescence plate reader (Infinite F200 PRO, Tecan). Glucose, lactate and intracellular ATP were evaluated in the presence and absence of rotenone (20 μM, 5 h) and 2-deox-glucose (2DG; 5 mM, 5 h) using commercial kits (Abcam) according to the manufacturer’s protocol. The results were normalized to the total protein content.

### Mitochondrial reducing activity

Mitochondrial reducing activity (MRA) was assessed in MiR-126-transfected MNSC cells and their parental counterparts as the reduction of resazurin based on the mitochondrial metabolic activity [15, 20]. Cells were incubated with resazurin (6 μM) and fluorescence intensity read at 0–240 min in a fluorescence plate reader (Infinite F200 PRO). The results were normalized to the total protein detected using the Bradford assay (Sigma).

### Colony-forming assay

MNSC cells and their MiR-126 plasmid-transfected counterparts were seeded in 0.35% low melting point agar overlaid with 0.7% low melting point agar in 24-well plates and cultured at 37 °C and 5% CO_2_ for 1 month. Every 7 days, 0.5 ml of fresh medium was added to each well, the number of colonies counted, and the size and the shape evaluated after 3 weeks. In addition, MNSC colonies in soft-agar were treated with exosomes from HUVECs (20 μg/ml) each week, and their size and shape evaluated after a 3-week treatment. Alternatively, MNSC cells (10^3^ cells per well in 24-well plate) were treated with the MiR-126 mimetic (100 nM), and colony formation was evaluated. Colonies were fixed with formalin (4.0% *v*/v), stained with crystal violet (0.5% *w*/*v*), and counted using a stereomicroscope.

### Triple co-culture model

A 3-μm trans-well insert (Costar 3452; Corning,) was first plated with 10^5^ cultured IMR90 cells in an inverted position. After 6 h of incubation, inserts were flipped over and placed into a six-well trans-well plate, where 3 × 10^5^ HUVEC cells were loaded on the other side of the insert and cultured for 24 h. This HUVEC-IMR90 pre-coated trans-well inserts were then placed into another six-well trans-well plate, where 2 × 10^5^ malignant nasal-septum carcinoma (MNSC) cells had been plated. Exosome-exposed HUVECs (50 μg/ml) were added to the triple co-culture system, and after 48 h of incubation MNSC cells were collected.

### Western blot analysis

Cells were lysed in the RIPA buffer containing Na3VO4 (1 mM) and protease inhibitors (1 μg/ml). The cell lysate proteins were separated using SDS-PAGE and transferred onto nitrocellulose membranes (Protran). After blocking with 5% non-fat milk in PBS-Tween (0.1%), the membranes were incubated with antibodies against IRS1 (Bethyl), phospho-p38 MAPK, p38-MAPK, phospho-ERK1/2 and ERK1/2 (all Cell Signaling). β-actin was used as a loading control. After incubation with the HRP-conjugated secondary IgG (Sigma), blots were developed using the ECL detection system (Pierce Biotechnology). The band intensities were visualized and quantified with ChemiDoc using the Quantity One software (BioRad Laboratories).

### Statistical analysis

Data are expressed as mean values ± standard deviation (SD). Comparisons among groups of data were performed using one-way analysis of variance (ANOVA), with Tukey post-hoc analysis. A *p*-value ≤0.05 was considered significant. All statistical analyses were performed using the SPSS software.

## Results

### MiR-126 level and epigenetic regulation

**Fig. 1:**
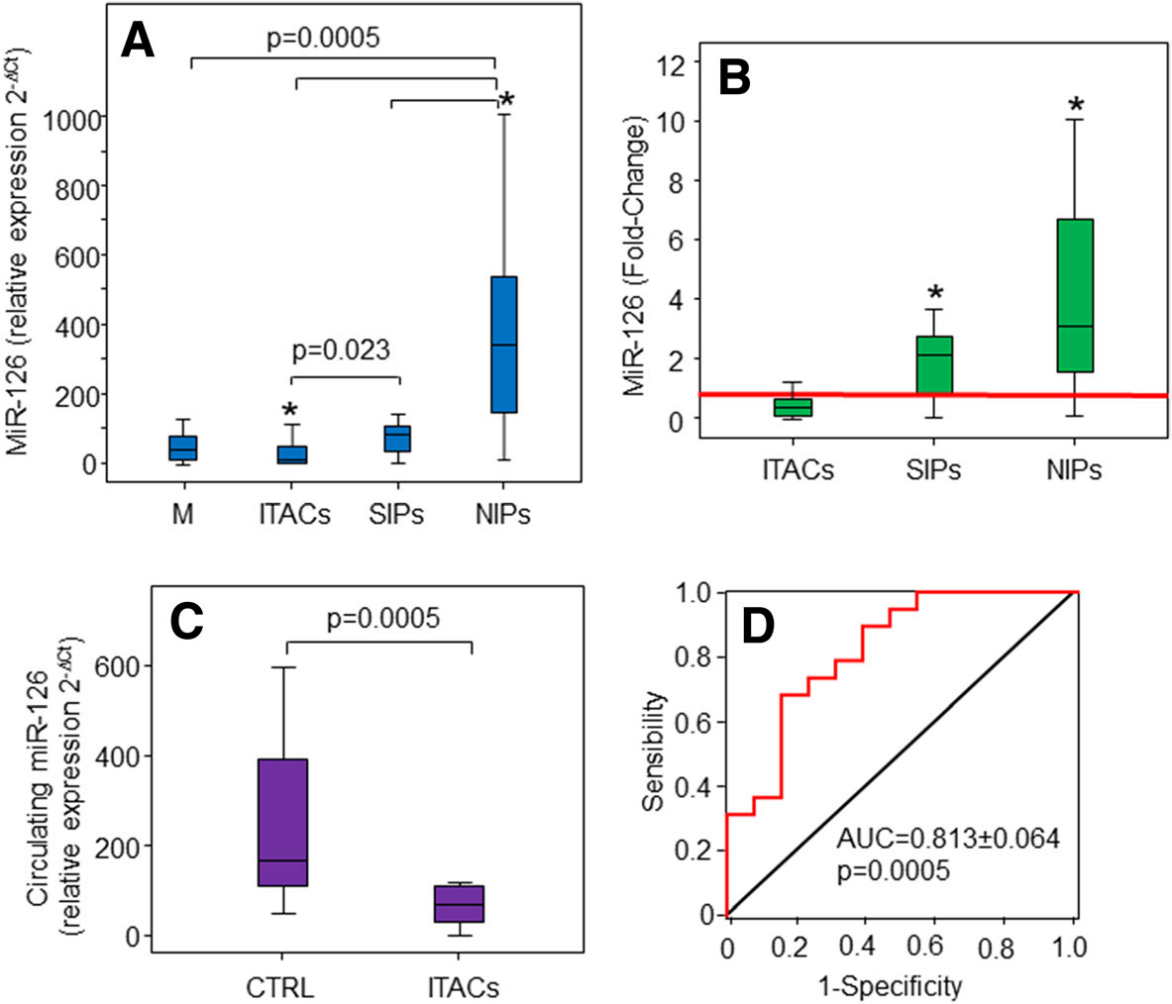
Levels of MiR-126 in tissues and serum of studied populations and its biomarker performance. Box-plots showing MiR-126 levels in biopsies of normal mucosa (M), Intestinal-type adenocarcinomas (ITACs), sinonasal inverted papillomas (SIPs) and nasal inflammatory polyps (NIPs) shown as relative expression (2^-∆Ct^) (**a**) or fold-change (**b**). **c** Relative expression of MiR-126 in serum of patients with ITACs and in healthy controls. **d** Receiver-operating curve (ROC) of serum MiR-126 to differentiate between ITAC patients and control subjects. MiR-126 yielded an AUC of 0.813 ± 0.064 with 80% sensitivity and 70% specificity in discriminating patients with SNC from healthy subjects, *p* < 0.0005. *Mucosa versus ITAC, SIP, and NIP groups, *p* < 0.05

There were no significant differences in age and gender among the studied groups, as documented in Table 1 summarising the demographic and pathological characteristics of the subjects. MiR-126 levels were significantly decreased in ITAC tissue (Fig. 1a), while MiR-126 in SIP and NIP tissues were strongly increased compared to the matched normal mucosa (Fig. 1a, b). Low level of MiR-126 was also found in serum of patients affected by ITACs (Fig. 1c), which significantly distinguished patients with ITACs from healthy controls with a sensitivity and specificity of 80% and 70% (Fig. 1d).

**Table 1:** Demographic and pathological characteristics of the study population

	Biopsy-samples			Serum-samples	
	ITACs (*n* = 23)	SIPs (*n* = 15)	NIPs (*n* = 20)	ITACs (*n* = 13)	CTRL (*n* = 19)
Age (years)	70.1 ± 11.6	57.4 ± 18.0	57.5 ± 15.4	66.2 ± 11.6	40.5 ± 11.4
Gender (M/F %)	91/9	67/33	85/15	92/8	84/16
Smoking (yes/no %)	–	–	–	58/42	47/53

*ITACs* Intestinal-type adenocarcinomas, *SIPs* sinonasal inverted papillomas, *NIPs* nasal inflammatory Polyps

**Fig. 2:**
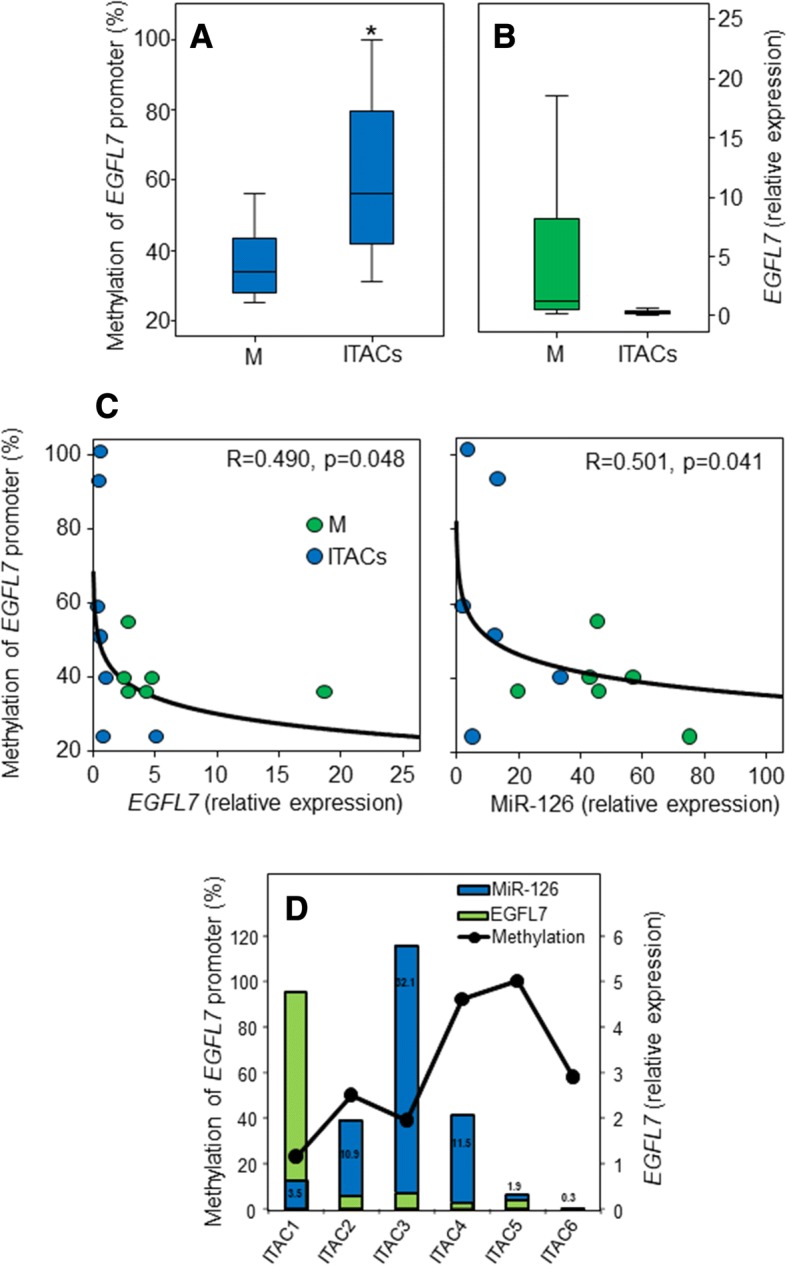
Epigenetic regulation of MiR-126 expression via changes in the methylation status of the host gene *EGFL7* T2 promoter. **a** Methylation level of the *EGFL7* promoter in biopsies of non-cancerous mucosa (M) and intestinal-type adenocarcinomas (ITACs). **b** Expression of *EGFL7* in biopsies of normal mucosa (M) and Intestinal-type adenocarcinomas (ITACs). **c** *EGFL7* gene (left-panel) and MiR-126 (right-panel) expression correlate with the methylation status of the *EGFL7* T2 promoter. MiR-126 and *EGFL7* expression was determined by qPCR and normalized to RNU6B RNA and G6PDH, respectively, in the clinical samples. **d** *EGFL7* gene expression and MiR-126 levels in cancer tissues in relation to the methylation status of the *EGFL7* T2 promoter. Methylation percentage was calculated using the bisulphite genomic sequencing results. *Mucosa versus ITACs, *p* < 0.05

The MiR-126 gene is located in an intron of a protein-coding gene, *EGFL7*, and its expression was previously reported to be closely correlated with the T2 *EGFL7* transcript [21]. To explore whether epigenetic regulation of the host *EGFL7* gene could directly affect MiR-126 expression, the T2 promoter methylation was investigated in malignant and adjacent non-cancerous tissues obtained from six surgical samples of ITAC patients. Highly methylated T2 promoter was found in ITAC patient tissues with down regulation of *EGFL7* gene expression (Fig. 2a, b). Correlation between EGFL7 promoter methylation and reduced expression of both MiR-126 and its host gene *EGFL7* was found (Fig. 2c). Despite the fact that *EGFL7* T2 promoter methylation above 40% resulted in a marked reduction of *EGFL7* gene expression in tumour tissues, no association with MiR-126 down-regulation was found (Fig. 2d). These results indicate that other factors in addition to epigenetic changes of the *EGFL7* T2 promoter are involved in MiR-126 expression during cancer progression.

### Ectopic expression of MiR-126 suppresses tumour growth and affects cell metabolism

Given that MiR-126 was found down-regulated in malignant tissues, we hypothesised that its increased expression could affect cancer development and progression. The development of sinonasal tumours, such as SNSCC and, especially ITAC, is strongly associated with occupational exposure, possibly through tumorigenic pathways of chronic inflammation. The SNSCC and ITAC have aneuploid genomes harbouring multiple genetic aberrations; we thus reasoned that both histotypes showed comparable response to miRNA treatment. According to the above, the MNSC cell line was used as an in vitro model for sinonasal tumours. Higher levels of MiR-126 following its overexpression and also transfection with the MiR mimetic resulted in a dose-dependent inhibition of MNSC cell growth (Additional file 1: Figure S1A). AntiMiR-126 reversed these effects, suggesting that the observed inhibition of cell proliferation is dependent on MiR-126 (Additional file 1: Figure S1B).

**Fig. 3:**
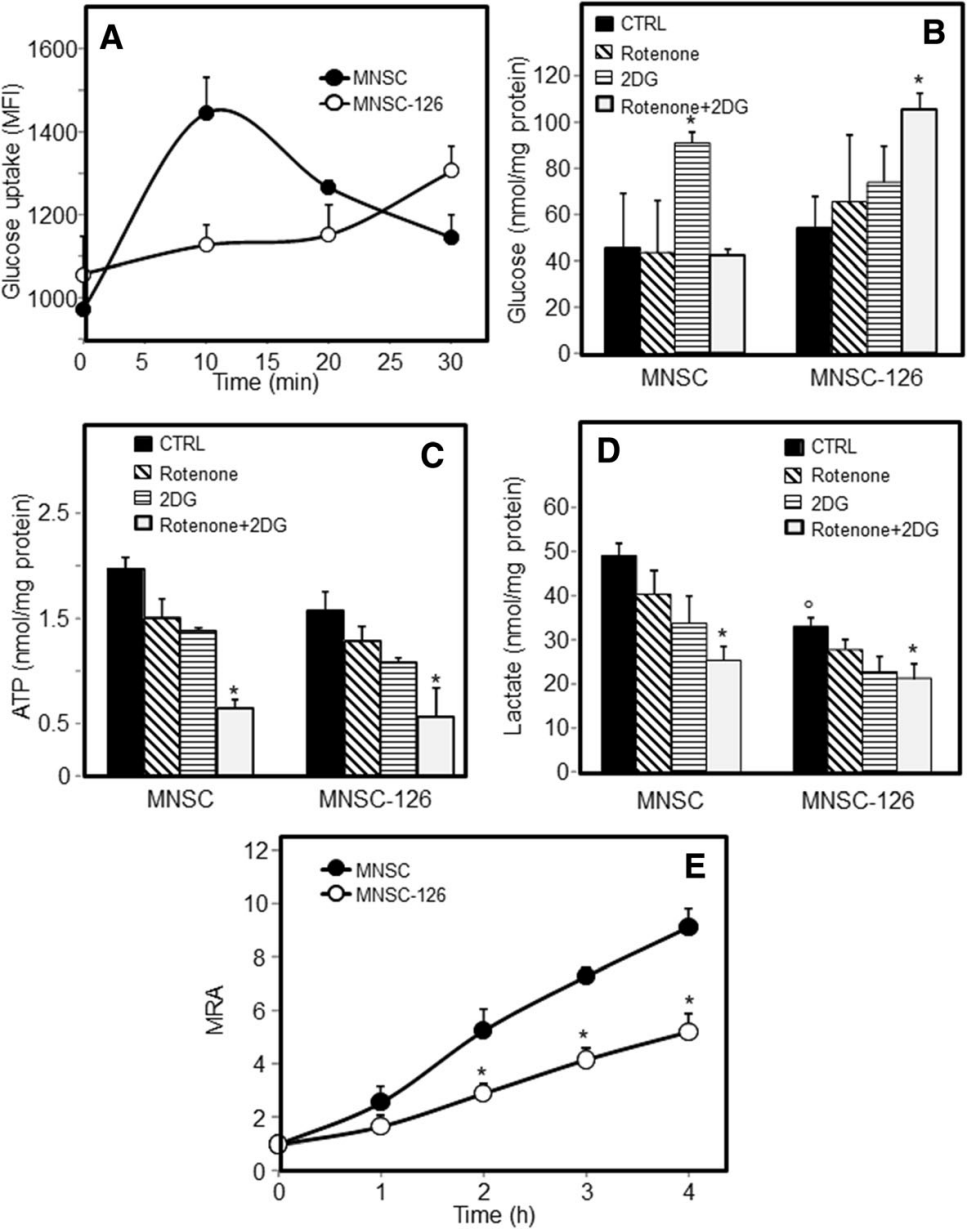
MiR-126 affects glucose homeostasis and bioenergetic profile. MiR-126-transfected malignant nasal-septum carcinoma (MNSC-126) cells and their counterparts (parental MNSC cells) were evaluated for glucose uptake (**a**) using 2-NBDG (50 μM) in low-glucose DMEM, expressed as mean fluorescent intensity (MFI). Intracellular levels of glucose (**b**), ATP (**c**) and lactate (**d**) were evaluated in the presence of rotenone (20 μM, 5 h) or 2DG (5 mM, 5 h). The mitochondrial reducing activity (MRA) was evaluated by means of the resazurin assay (**e**). The data shown are mean values ± S.D. derived from three independent experiments. The symbol “*” indicates significant differences compared with control; the symbol “°” compared with parental cells

Loss of malignancy was also observed in MiR-126-transfected MNSC cells. Ectopic expression of MiR-126 resulted in inhibition of colony formation, which was associated with metabolic changes. Glucose uptake and intracellular glucose, ATP and lactate were evaluated by inhibiting oxidative phosphorylation (OXPHOS) with rotenone or glycolysis with 2DG. MiR-126 alters glucose homeostasis by inhibiting glucose uptake and shifting the metabolism from glycolysis to OXPHOS. Differently to parental cell, where glucose is mainly metabolised by glycolysis, glucose was found to be processed via both OXPHOS and glycolysis in MiR-126-transfected MNSC cells resulting in lower ATP and lactate production associated with low rate of mitochondrial reducing activity (Fig. 3a, e). The metabolic shift was associated with morphological changes, acid vesicle formation and accumulation of lipid droplets (LDs) (Additional file 2: Figure S2).

### Exosomes with MiR-126 reduce cell growth and suppress malignancy

**Fig. 4:**
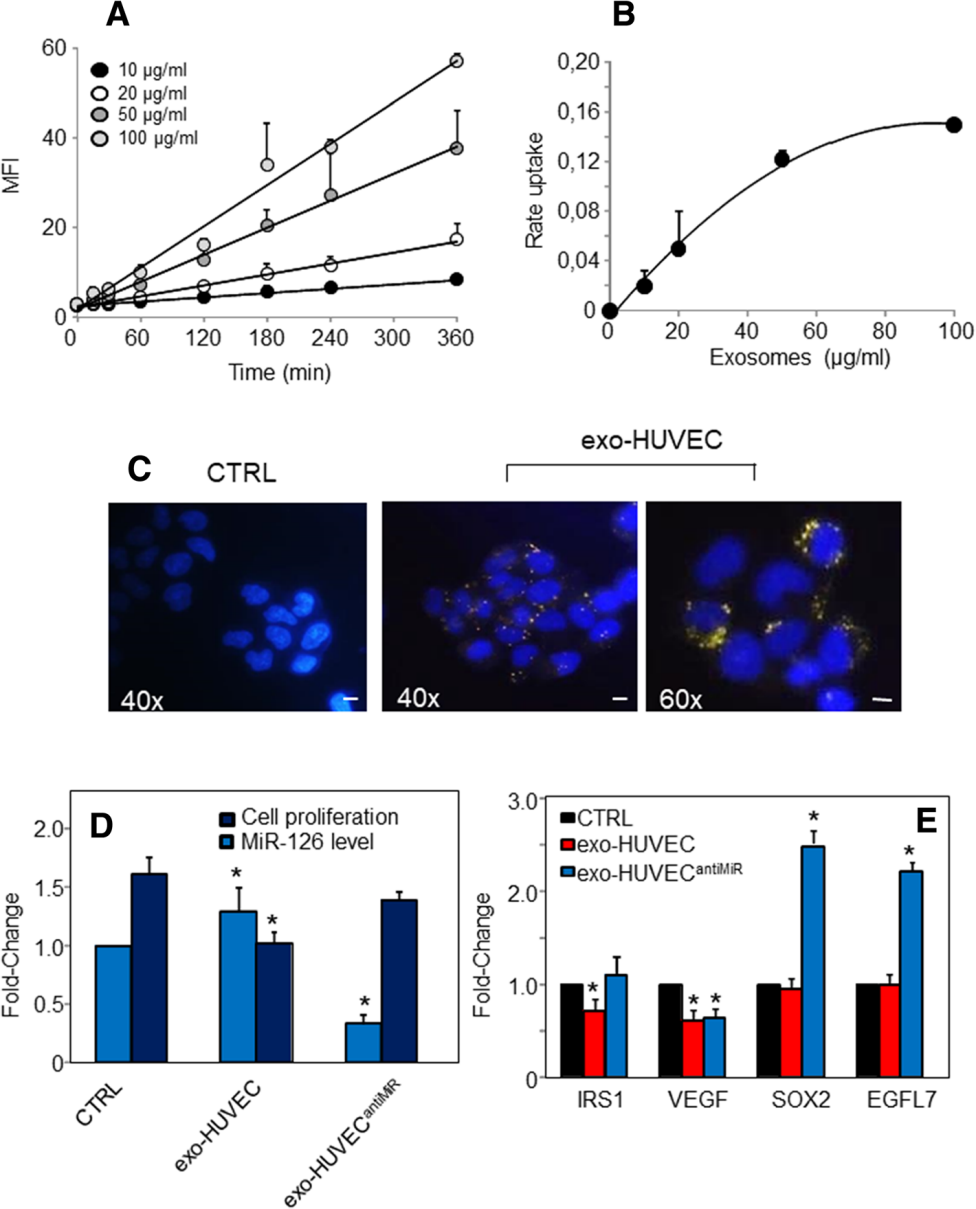
Exosomal transfer of MiR-126 from endothelial cells inhibits proliferation of malignant nasal-septum carcinoma (MNSC) cells. **a** Temporal and dose-dependent kinetics of HUVEC exosome uptake in MNSC cells, and (**b**) dose-response curve at 360 min. Exosomes (exo) were isolated from HUVECs grown in the medium supplemented with exosome-free serum. Isolated exosomes were stained with the lipophilic dye PKH-26 and extensively washed with two subsequent ultracentrifugation spins. Next, MNSC cells were incubated over time with PKH-26-labelled exosomes at different concentrations (μg/ml). Cell were then washed, trypsinized and their fluorescence analysed by flow cytometry, and the uptake was expressed as mean fluorescence intensity (MFI). **c** Representative fluorescence image of uptake of exosomes derived from HUVECs by MNSC cells. MNSC cells, cultured in exosome-depleted serum, were incubated with PKH-26 -labelled exosomes (20 μg/ml) from HUVECs for 4 h and their internalization visualized by fluorescent microscope (Zeiss, Axiocam MRc5; magnification 40-60×). The scale bar for all images equals 100 μm. **d** MiR-126 levels and cell proliferation of MNSC cells evaluated by the MTT assay after incubated with exosomes (20 μg/ml) isolated from HUVECs in the presence or absence of antiMiR. **e** Expression of MiR-126 targets IRS1, VEGF, SOX2, and EGFL7 after incubation of MNSC cells with exosomes isolated from HUVECs in the presence or absence of antiMiR. The data shown are mean values ± S.D. derived from three independent experiments. The symbol “*” denotes significant differences between non treated cells (CTRL) and exo-HUVEC treated cells with and without antiMiR, *p* < 0.05

Exosomal transfer of MiR-126 may have functional relevance. Endothelial cells release exosomes enriched in MiR-126 that are taken up by ECs themselves (paracrine signalling) or that translocate to other compartments to modulate the downstream intercellular signalling mediators [22, 23]. To evaluate the uptake of HUVEC-derived exosome by MNSC cells, a quantitative flow cytometric assay was performed. Fluorophore-labelled exosomes from HUVECs were administered at increasing concentration, and exosomal uptake by MNSC recipient cells was evaluated over time. As shown in Fig. 4a, uptake of exosomes was linear for up to 360 min and 100 μg/ml of exosome protein. Subsequent transport studies were carried out using the incubation time of 360 min. MNSC cells took up EC-derived PKH-67-labelled exosomes in a time- and concentration-dependent manner (Fig. 4a, b). As shown by the kinetic analysis, the uptake was rapid, and exosome internalization was visualized as punctuate green fluorescence (Fig. 4c). Next, the biological effect of exosome-delivered MiR-126 in cancer was investigated by treating MNSC cells with exosomes derived from HUVECs, and MiR-126 levels and cell proliferation were evaluated. As shown in Fig. 4d, MiR-126 was up-regulated compared to untreated MNSC cells, which was associated with inhibition of cell proliferation. AntiMiR transfection resulted in MiR-126 down-regulation, with subsequent increased cell proliferation, pointing to a MiR-126-specific effect. We found that exosomal MiR-126 inhibited the expression of its gene targets, such as insulin receptor substrate-1 (IRS1) and VEGF. The sex-determining region Y-box 2 (SOX2) and *EGFL7* were not affected by MiR-126 derived from HUVEC exosomes, whose expression was markedly induced by anti-MiR, supporting the notion that both targets were regulated by MiR-126 acting via a negative feedback mechanism (Fig. 4e).

**Fig. 5:**
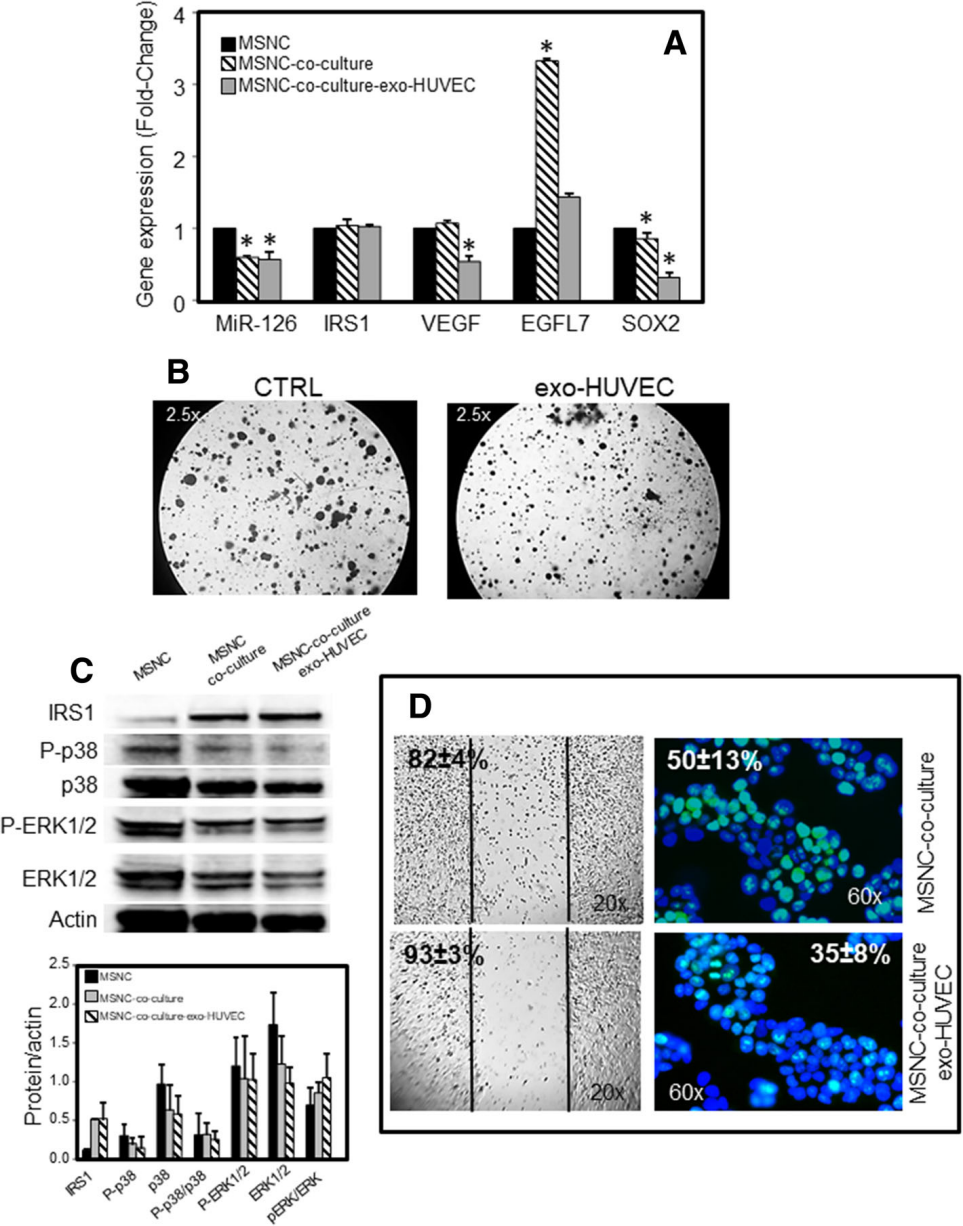
Exosome-delivered MiR-126 from endothelial cells as a treatment of malignant nasal-septum carcinoma (MNSC) cells. **a** Cancer cell-stromal cell cross talk. Triple co-culture was performed by layering fibroblasts (IMR-90) and endothelial cells (HUVEC) on two opposite surfaces of trans-well inserts, and MNSC cells were cultured at the bottom of the trans-well. The co-culture was treated with exosomes from HUVEC (exo-HUVEC, 50 μg/ml), and after 2 days of treatment MiR-126 level and the expression of its gene targets were evaluated in MNSC cells. **b** Effect of exosomes from endothelial cells on colony-forming activity in MNSC cells. The images shown represent 3 months of treatment (magnification 2.5×). **c** Immunoblot evaluation of MAPK signalling. MNSC cells in triple co-culture were treated with exo-HUVEC (20 μg/ml), and after 2 days of incubation evaluated for the expression of IRS1, pERK1/2, ERK1/2, p-p38, p38 (upper-panel). Densitometry evaluation of the bands related to the level of actin (down-panel). **d** Representative images of wound healing mobility assay (left-panel) and proliferative index Ki67 staining (right-panel). MNSC cells in triple co-culture were treated with exo-HUVEC (20 μg/ml), and after incubation evaluated for wound-healing mobility percentage and percentage of Ki67 positive cells. All experiments were carried out in triplicate. The symbol “*” denotes significant differences between non treated cells (CTRL) and exo-HUVEC treated cells, *p* < 0.05

To get additional support for the function of MiR-126 as a tumour suppressor, a triple co-culture model was used. MNSC cells were co-cultured with fibroblasts (IMR-90) and HUVECs, and treated with exosomes derived from HUVECs. After 48 h, MNSC cells were collected and analysed for MiR-126 and expression of its target genes. Cell migration and proliferation were also evaluated in the triple co-culture model after incubation with exosomes from HUVECs. Downregulation of MiR-126 and up-regulation of *EGFL7* were observed as a result of the co-culture. Similar to this, decreased expression of MiR-126 was found following exposure to exosomes derived from HUVECs. However, this treatment resulted in suppression of VEGF and SOX2 gene expression (Fig. 5a). As shown in Fig. 5b, MiR-126 delivered via HUVEC-derived exosomes significantly inhibited colony formation and growth in soft agar, markers of a malignant status. Incubation with exosomes induced a slight reduction of the MAPK/ERK pathway, which was associated with inhibition of cell migration and proliferation (Fig. 5c, d).

## Discussion

In the present study we found that the angiogenic MiR-126 may distinguish between malignant and benign forms, such as SIP and NIP (cf Fig. 1). In particular, MiR-126, expressed at low levels in ITACs due to changes in the methylation status of the host gene *EGFL7* T2 promoter (cf Fig. 2), was found up-regulated in SIP and even more in NIP. Methylation-associated regulation of MiR-126 and its host gene *EGFL7* has been described in other tumours [24–26]. Although epigenetic modification is a crucial mechanism for controlling the expression of miR-126, we did not find a strict relationship between MiR-126 expression and methylation status of the host EGFL7 gene in SNC tissues (cf Fig. 2). There are other factors linked to cancer metabolism or exosomal delivery from neighbouring cells that may affect MiR-126 expression [14, 22, 23]. It was suggested that a cancer stroma cross-talk induced repression of MiR-126 to facilitate angiogenesis and invasion [27]. MiR-126 is enriched in ECs, modulating the level of diverse transcripts that control angiogenesis. Expression analysis documents increased levels of MiR-126 in highly vascularized tissues, such as the heart, liver, and lung. On the other hand, MiR-126 has been shown to be a negative modulator of angiogenesis in the eye [28]. There is evidence that MiR-126 contributes to vascular homeostasis by inhibiting angiogenesis and maintaining the quiescent endothelial phenotype associated with increased vascular integrity and inhibited proliferation and motility. MiR-126 up-regulation activates endothelial progenitor cells (EPCs) and ECs in the case of vascular injury and/or hypoxia, contributing to vascular healing and formation of new blood vessels [29]. High level of MiR-126 induces angiogenesis in non-malignant tissues by a mechanism that involves MiR-126-induced inhibition of the tumour suppressor SPRED1 [30]. Conversely, low levels of MiR-126 contribute to the deregulation of blood vessel formation in tumours, probably by enhancing VEGF expression [31]. These dual pro- and anti-angiogenic properties make MiR-126 a plausible biomarker to detect transition from the benign to the malignant phenotype. This is the case of SIP, which is characterized by a relatively strong potential for local tissue destruction, high rate of recurrence, and a risk of carcinomas. The follow-up of patients is critical to diagnose local relapse, which often occurs as an early event. The serious nature of this pathology is linked to its association with carcinomas, which may be diagnosed at the onset or the recurrence during the follow-up. It is therefore imperative to diagnose recurrence of the pathology in early stages in order to initiate early treatment, especially in the case of SIP-associated carcinomas [32, 33].

As previously reported, low levels of MiR-126 in cancer [12], including SNC, highlight its role in tumour formation and development. In this context, restoration of MiR-126 induced metabolic changes and inhibited cell growth and tumorigenesis of cultured MNSC cells (cf Additional file 1: Figure S1 and Additional file 2: Figure S2). Cancer cells prefer glycolysis and circumvent OXPHOS for ATP generation; consistent with this, we found that MiR-126 inhibited glucose uptake and glycolysis, thus restoring OXPHOS associated with a decrease in the mitochondrial redox activity (cf Fig. 3). Taking into account the possibility that exosomes released by cancer cells present a major factor driving the cancer phenotype, we can hypothesize that exosomes from normal cells can be used as a vehicle for delivery of anti-tumour factors. Since MiR-126 is secreted by ECs by means of exosomes and since it can be readily internalised by recipient cells, we used exosomes as carriers to deliver MiR-126 to cancer cells. Exosomal MiR-126 derived from HUVECs was found to interact with MNSC cells, leading to increased MiR-126 levels, whereby inducing anti-tumour responses such as inhibition of cell proliferation and target gene modulation including IRS1 and VEGF (cf Fig. 4).

To mimic the tumour stroma, a triple co-culture model was performed. As previously reported [27], we found that down-regulation of MiR-126 was related to cancer stroma. This down-regulation can be attributed to a cross-talk among cancer cells and the adjacent fibroblasts and angiogenic endothelial cells. Thus, targeting MiR-126 can be an innovative means to normalize the aberrant vasculature in cancer environment. In this context, exosomal MiR-126 significantly reduced VEGF gene expression associated with reduced colony formation, and inhibition of cell migration and proliferation (cf Fig. 5).

Involvement of MiR-126 in cancer biology is not limited to modulation of angiogenesis. Our results indicate that this miRNA plays a role in cancer by altering several cellular mechanisms of cancer pathogenesis. Exosomes containing miRNAs represent a promising new therapeutic approach, since they play an important natural role in cellular processes combined with high stability, tissue-specific expression, and secretion into body fluids. The half-life of exosomes in the circulation is greater than that of liposomes due to their endogenous origin and unique surface composition. This enables them to specifically bind to recipient cell receptors, pointing to the possibility of utilising exosomes that specifically target a relevant cell type. Moreover, exosomes are not immunogenic and can carry diverse cargo that will not be ‘destroyed’ prior to delivery.

## Conclusions

Our report suggests a potential role of circulating MiR-126 as a biomarker to differentiate malignant types from benign forms of nasal neoplastic pathologies. The transfer of MiR-126 mediated by exosomes impacts on protein regulation in acceptor cells, resulting in altered cellular phenotypes. Thus, paracrine transfer of miRNAs, as epitomised here by MiR-126, presents a new approach to develop microRNA-based therapies for neoplastic pathologies as well as improved diagnostic tools.

## Additional files

Additional file 1: **Figure S1.** MiR-126 inhibits proliferation of cultured malignant nasal-septum carcinoma (MNSC) cells. (**A**) MNSC cells were treated with MiR-126 mimetic at increasing concentration, and MiR-126 content (insert) and cell proliferation were evaluated using the MTT assay. (**B**) Cells were transfected with MiR-126 mimic (100 nM), plasmid MiR-126, or antisense MiR-126 (antiMiR), and evaluated for their growth using the MTT assay. The data shown are mean values ± S.D. derived from three independent experiments. (TIF 886 kb)

Additional file 2: **Figure S2.** MiR-126 suppresses the malignant phenotype of malignant nasal-septum carcinoma. (**A**) Colony-forming activity was evaluated using cells transfected with MiR-126 (left panel) or MiR-126 mimetic (100 nM, right panel). (**B**) Morphology and metabolic stress were evaluated as formation of acid vesicle (acridine orange staining) and lipid droplets (LDs, Red-Oil O-staining) in plasmid-MiR-126 transfected MNSC cells and in their parental counterparts. The scale bar for all images equals 10 μm. The images (representative of three independent experiments) visualized by fluorescent microscopy (Axiocam MRc5, Zeiss, magnification 20×, 40× and 60×). (TIF 2484 kb)

## Abbreviations

AO: acridine orange
AVs: Acid vesicles
CDX2: homeobox transcription factor 2
CK: Cytokeratin
ECs: Endothelial cells
*EGFL7*: EGF-like domain-containing protein 7
EPCs: Endothelial progenitor cells
FFPE: Formalin-fixed, paraffin-embedded
HUVECs: Human umbilical vein endothelial cells
IRS1: Insulin receptor substrate-1
ITACs: Intestinal-type adenocarcinomas
MiRs: MicroRNAs
MNSC: Malignant naso-sinusal cancer
MTT: 3-(4,5-dimethylthiazol-2-yl)-2,5-diphenyltetrazolium bromide
NIPs: Inflammatory polyps
SIPs: Sinonasal-inverted papillomas
SNCs: Sinonasal cancers
SNSCC: Squamous cell carcinoma
SOX2: Sex-determining region Y-box 2
VEGF: Vascular endothelial growth factor
