# Supplementary figures and images for: MiR-126 in intestinal-type sinonasal adenocarcinomas: exosomal transfer of MiR-126 promotes anti-tumour responses
Source: BMC Cancer. 2018 Sep 17;18:896. doi: 10.1186/s12885-018-4801-z (PMC6142309; doi:10.1186/s12885-018-4801-z)

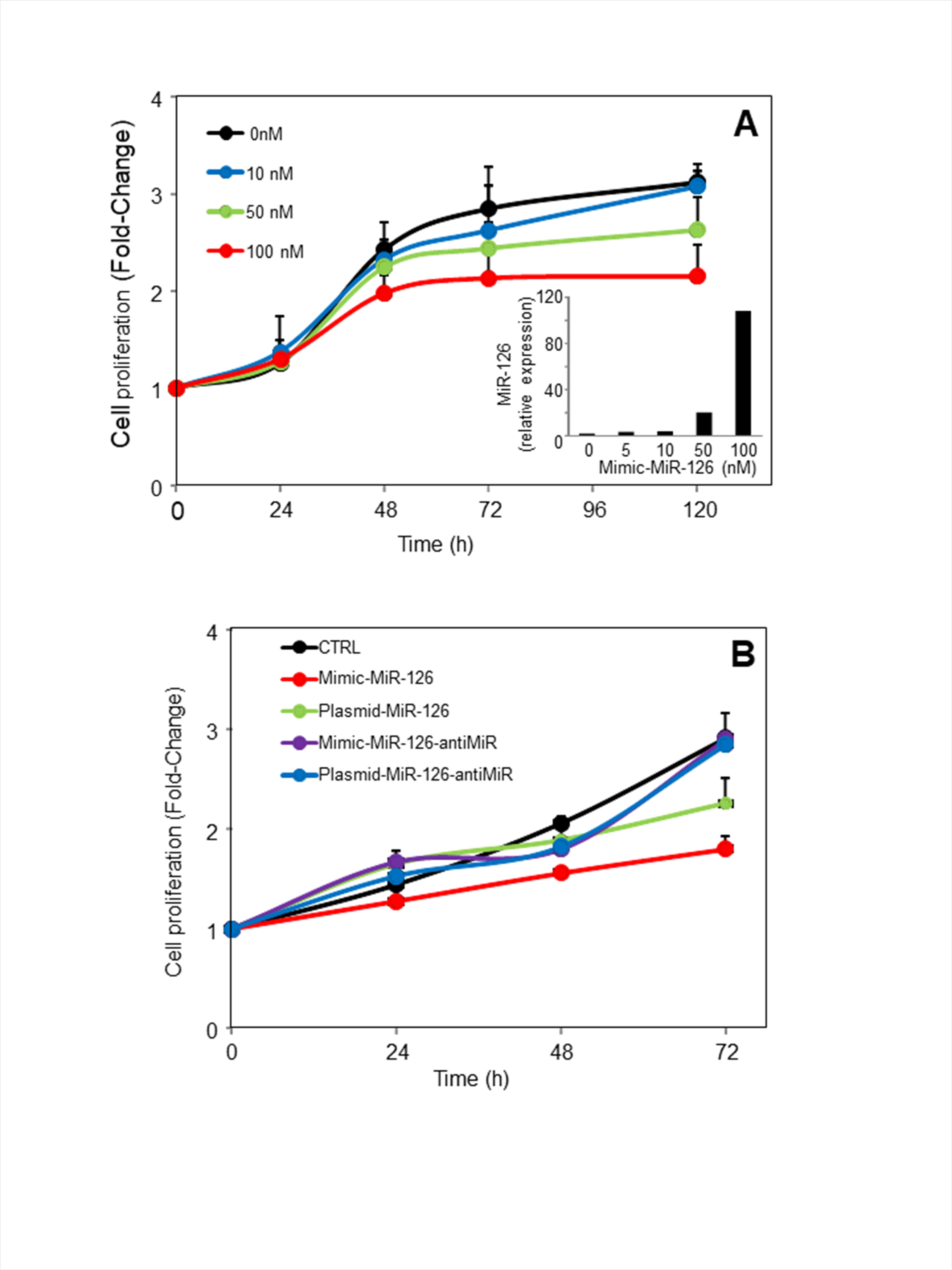

Supplement: Supplementary file 1 — Figure S1. MiR-126 inhibits proliferation of cultured malignant nasal-septum carcinoma (MNSC) cells. (A) MNSC cells were treated with MiR-126 mimetic at increasing concentration, and MiR-126 content (insert) and cell proliferation were evaluated using the MTT assay. (B) Cells were transfected with MiR-126 mimic (100 nM), plasmid MiR-126, or antisense MiR-126 (antiMiR), and evaluated for their growth using the MTT assay. The data shown are mean values ± S.D. derived from three independent experiments. (TIF 886 kb) [file 12885_2018_4801_MOESM1_ESM.tif]

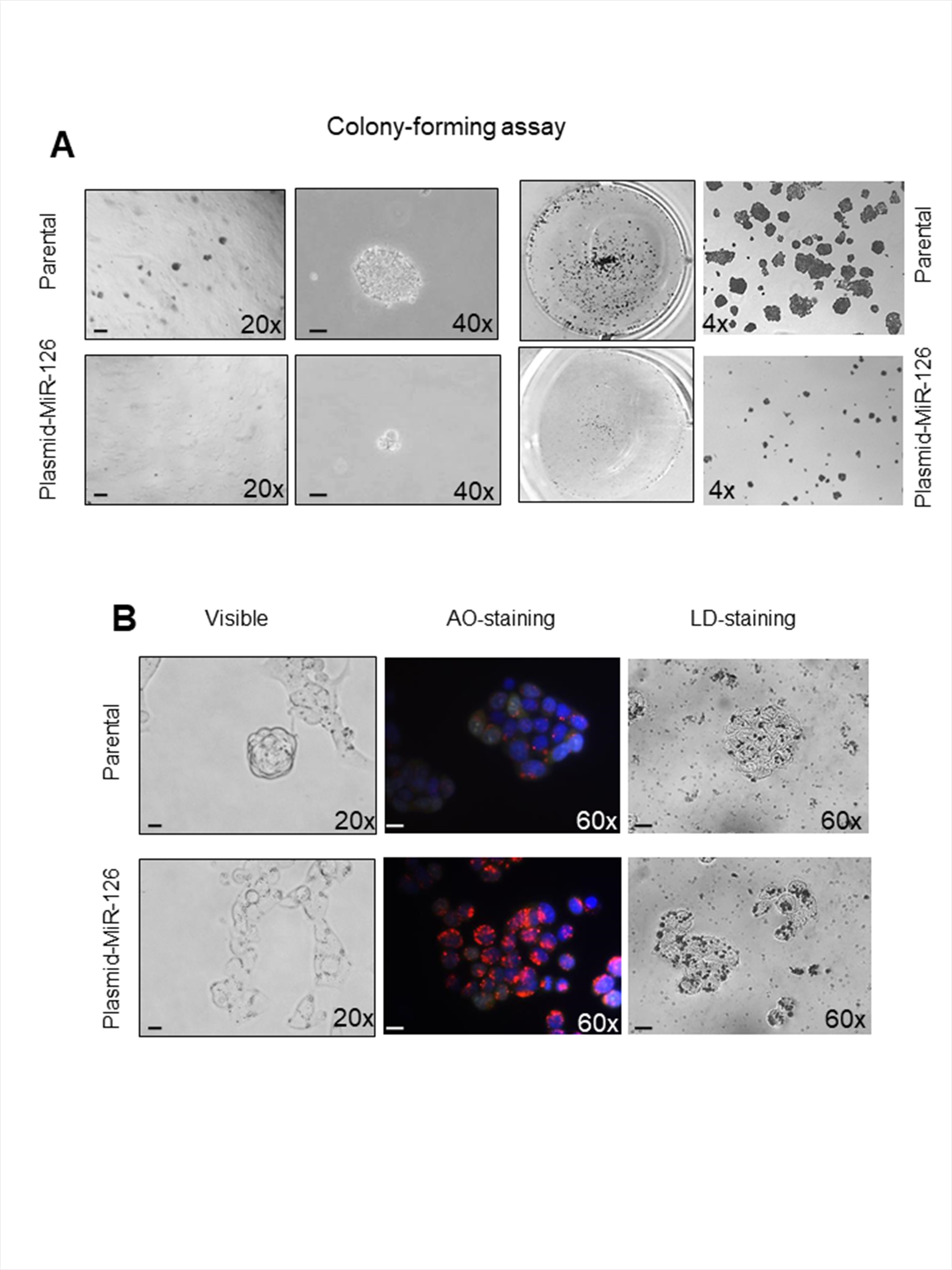

Supplement: Supplementary file 2 — Figure S2. MiR-126 suppresses the malignant phenotype of malignant nasal-septum carcinoma. (A) Colony-forming activity was evaluated using cells transfected with MiR-126 (left panel) or MiR-126 mimetic (100 nM, right panel). (B) Morphology and metabolic stress were evaluated as formation of acid vesicle (acridine orange staining) and lipid droplets (LDs, Red-Oil O-staining) in plasmid-MiR-126 transfected MNSC cells and in their parental counterparts. The scale bar for all images equals 10 μm. The images (representative of three independent experiments) visualized by fluorescent microscopy (Axiocam MRc5, Zeiss, magnification 20×, 40× and 60×). (TIF 2484 kb) [file 12885_2018_4801_MOESM2_ESM.tif]
